# 2479: Validating acute urinary retention using diagnosis and procedure codes

**DOI:** 10.1017/cts.2017.282

**Published:** 2018-05-10

**Authors:** Scott Martin Vouri, Seth Strope, Margaret Olsen

## Abstract

OBJECTIVES/SPECIFIC AIMS: We evaluated the accuracy of diagnosis and procedure codes to identify acute urinary retention (AUR) due to lack of existing validation studies. METHODS/STUDY POPULATION: We performed a cross-sectional validation study at a single medical institution in the emergency department (ED) and outpatient Urology Clinic in men ≥45 years. International Classification of Diseases, Ninth Revision, Clinical Modification (ICD-9-CM) diagnosis codes 788.20, 788.21, 788.29 for urinary retention and Current Procedural Terminology, Fourth Edition (CPT-4) codes 51701, 51702, 51703 for urinary catheterization were used to identify men with potential AUR. Four algorithms using ICD-9-CM and CPT-4 codes were compared against medical record review. Sensitivity, specificity, positive predictive value, negative predictive value, and area under the curve were calculated for both the ED and Urology Clinic. RESULTS/ANTICIPATED RESULTS: A total of 333 treated and released men in the ED were identified using facility billing data, and 245 men using physician billing data in the Urology Clinic were identified using the codes for urinary retention or urinary catheterization. Of the 4 algorithms, any ICD-9-CM diagnosis code for urinary retention was the preferred algorithm with a sensitivity and specificity of 0.95 and 0.91 using ED facility billing data and a sensitivity and specificity of 0.95 and 0.58 using Urology physician billing data. DISCUSSION/SIGNIFICANCE OF IMPACT: Use of the ICD-9-CM diagnosis codes for urinary retention performed well at identifying AUR in the ED. This study provides justification to use urinary retention diagnosis codes (specifically 788.20 and 788.29) in future studies to identify AUR using administrative data, especially in the ED.

